# Resistance Spectrum Analysis and Breeding Utilization of Rice Blast Resistance Gene *Pigm-1*

**DOI:** 10.3390/plants14040535

**Published:** 2025-02-10

**Authors:** Yidan Jin, Niqing He, Zhaoping Cheng, Shaojun Lin, Fenghuang Huang, Wenxiao Wang, Qingshun Q. Li, Dewei Yang

**Affiliations:** 1 Institute of Rice, Fujian Academy of Agricultural Sciences, Fuzhou 350018, China; jyd0121@163.com (Y.J.); heniqing430@163.com (N.H.); chengzhaoping@126.com (Z.C.); shaojunlin1993@163.com (S.L.); hhf13305022536@163.com (F.H.); wenxiaowang98@163.com (W.W.); qqli@westernu.edu (Q.Q.L.); 2 College of Agriculture, Fujian Agriculture and Forestry University, Fuzhou 350002, China; 3 Biomedical Sciences, College of Dental Medicine, Western University of Health Sciences, Pomona, CA 91766, USA

**Keywords:** rice, blast disease, *Pigm-1*, resistance spectrum, breeding utilization

## Abstract

Rice blast is one of the most important diseases of rice, causing significant economic losses to agricultural production. A new gene, *Pigm-1*, which is allelic to *Pigm*, was cloned from Shuangkang 77009 using map based cloning. However, it is unclear whether there is a difference in the resistance spectrum between *Pigm* and *Pigm-1*. In this study, using 195 rice blast isolates collected from different areas of the Fujian Province, the *Pigm-1* and *Pigm* single gene lines were inoculated to test their resistance. There was only one blast fungus JL-37 that showed a differential response in *Pigm* and *Pigm-1* single gene lines, while the remaining 194 showed no difference. To further explore the application range of *Pigm-1*, the resistant rice R20-4 containing *Pigm-1* was used as the donor, and a sensitive sticky rice S19-118 was used as the receptor. The hybrid F_1_ was first backcrossed with S19-118 using a molecular marker-assisted selection breeding method, and a strain containing the *Pigm-1* gene was selected to continue to backcross with S19-118 until BC_3_F_1_. A new blast resistance rice material, Xiannuo 23, containing *Pigm-1* was developed and confirmed by laboratory and field tests. This material can be broadly used for the future breeding of rice blast resistant cultivars to reduce the loss of rice production.

## 1. Introduction

Rice (*Oryza sativa*) is one of the most important food crops in the world, with about half of the world’s population using it as their staple food [1]. Rice blast is a worldwide disease caused by *Magnaporthe oryzae* [2], which can cause at least 10% yield loss of rice every year. In severe cases, the yield reduction in rice can be as high as 40% to 50%, or even total loss [3,4]. At present, the main method of rice blast control is chemical application, but excessive use of chemical agents has caused serious damage to the environment [5,6]. Under normal circumstances, new varieties that are resistant to rice blast will gradually lose resistance after 3 to 5 years of field production, mainly due to the narrow spectrum of resistance genes carried by cultivars. The physiological variants of rice blast have strong variability, leading to rapid disease development under suitable environmental conditions [7]. Rice blast resistance breeding is still the most effective and equitable means to limit the losses in rice production [8]. Therefore, it is of great significance to explore more blast resistance genes and analyze their resistance spectra, so as to create new resistant cultivars for ensuring food security [9].

So far, over 100 rice blast resistance loci have been genetically characterized, and more than 50 blast resistance genes have been identified [5,8]. These genes are distributed on different chromosomes of rice. At present, several major genes such as *Pi2*, *Pi9* (*Pi9-type-3/4/5/6/9/10/11*), *Piz-t* (*Pizh*) and *Pigm* (*Pi50*) have been cloned at the *Piz* locus on chromosome 6 of rice [10,11,12]. Using molecular marker-assisted selection, the resistant rice varieties can be produced by introducing the broad-spectrum blast resistance genes into susceptible genotypes [13]. Different alleles of the blast resistance gene at one locus have different species-specific spectra [8]. For example, seven alleles with different resistance profiles were identified at *Pik* resistance sites [14,15]. Therefore, it is particularly important to explore and isolate more resistance genes and their alleles. Previously, our research team cloned a broad-spectrum blast resistance gene *Pigm-1* from the disease-resistant resource material Shuangkang 77009 by map based cloning. This gene is a new allele of *Pigm*, which is different from *PigmR* by 3 bases [16]. In recent years, through molecular breeding, researchers have created a series of new restorer line materials containing *Pigm* and its allele *Pigm-1* [16,17,18].

*Pigm-1* and *Pigm* are alleles and differ from each other by only a few bases. It is not clear whether their resistance spectra are different. On the other hand, although we have used *Pigm-1* to create a series of new disease-resistant materials, there are few reports on the creation of new special rice materials containing *Pigm-1*. In this study, different blast fungi were isolated from Shanghang County, Jianyang County and Jiangle County, Fujian Province, and inoculated with *Pigm-1* and *Pigm* single gene lines in order to find the resistance spectrum of these resistance genes. At the same time, to further explore the application range of *Pigm-1*, this study created a new disease-resistant glutinous rice material containing *Pigm-1* through molecular breeding methods, and provided excellent disease-resistant new materials for specialty rice breeding.

## 2. Results

### 2.1. Creation of Single Gene Lines Containing Pigm-1 and Pigm

**Figure 1:**
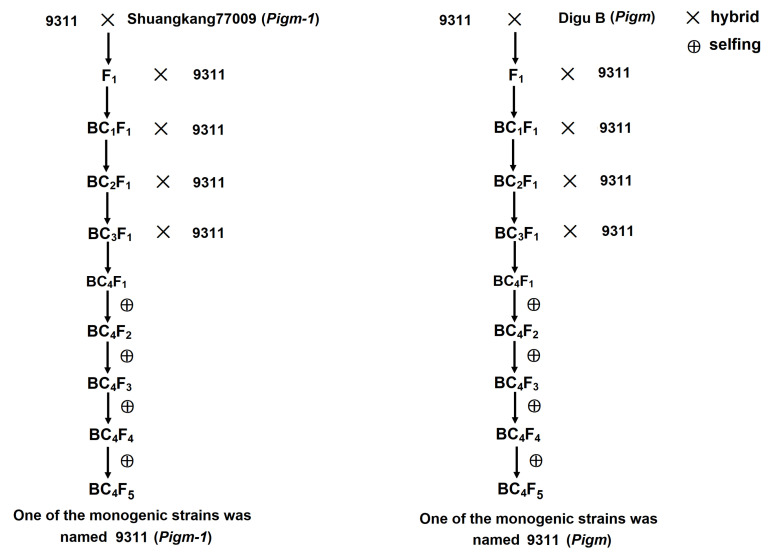
Construction roadmap of *Pigm-1* and *Pigm* monogenic lines. BC, backcross; F, familial.

To obtain *Pigm-1* and *Pigm* single gene lines, the parent Shuangkang 77009 containing *Pigm-1* and the parent Digu B containing *Pigm* were used as the donors, and 9311 as the acceptor. The F_1_ plants were generated from Shuangkang 77009 and Digu B as female and 9311 as male. The F_1_ plants were backcrossed with 9311 to produce the BC_1_F_1_ generation. The BC_1_F_1_ plants were backcrossed to 9311 to produce BC_2_F_1_, and the BC_2_F_1_ plants were backcrossed to 9311 to produce BC_3_F_1_, and then further backcrossed all the way to BC_4_F_1_. At the same time, *Pigm-1* and the molecular marker corresponding to *Pigm* were detected in each backcross. From BC_4_F_1_ to BC_4_F_5_, molecular markers were tracked for detection in each generation and, finally, *Pigm-1* and *Pigm* single gene lines were obtained, named 9311 (*Pigm-1*) and 9311 (*Pigm*), respectively (Figure 1).

### 2.2. Analysis of the Main Agronomic Traits of Pigm-1 and Pigm Single Gene Lines

To further analyze the changes in other agronomic traits of the improved line after the integration of blast resistance, we investigated the agronomic traits of 9311 (*Pigm-1*), 9311 (*Pigm*) and 9311 at maturity. The results showed that there was no difference between 9311 and 9311 (*Pigm*) in main agronomic traits, including plant height, panicle length, spikelets per panicle, seed setting rate, effective panicle number, grain length, grain width and 1000-grain weight. However, there was no difference in agronomic traits between 9311 and monogenic line 9311 (*Pigm-1*) except plant height, panicle length and spikelets per panicle (Table 1). These results indicate that the genetic background of single-gene line 9311 (*Pigm-1*) and 9311 (*Pigm*) basically restore the receptor parent 9311.

### 2.3. Analysis of Single Gene Line Pigm-1 and Pigm Blast Resistance Spectrum

**Figure 2:**
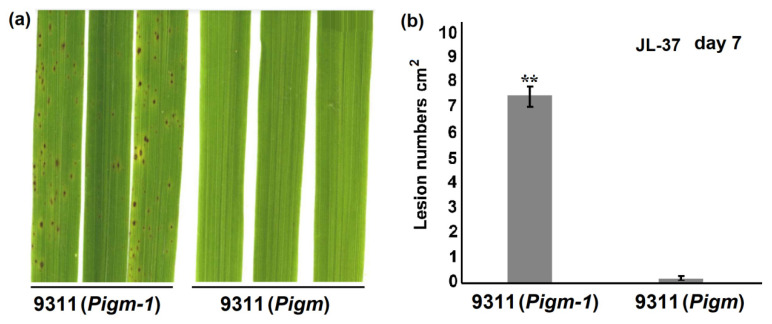
The disease symptoms of the rice cultivars 9311 (*Pigm-1*) and 9311 (*Pigm*) infected by blast fungi. (**a**) Blast resistance of 9311 (*Pigm-1*) and 9311 (*Pigm*) plants using spraying inoculation in a greenhouse. Representative leaves obtained 7 days after inoculation with blast strain JL-37. (**b**) Lesion numbers cm^2^ on the rice leaves (M ± SD, *n* > 10 leaves) in (**a**), Asterisk (**) indicates statistical significance (*p* < 0.01) determined by Student’s *t* test.

These 195 isolates were used to inoculate 9311 (*Pigm-1*), 9311 (*Pigm*) and 9311. The results showed that the three materials were resistant to 5 blast isolates (JL-12, SH-17, SH-26, SH-68 and JY-7). However, 9311 (*Pigm-1*) and 9311 (*Pigm*) were resistant, but 9311 was susceptible, to other 189 isolates. Only one blast strain JL-37 showed differential virulence where 9311 (*Pigm*) shows resistance, but 9311 (*Pigm-1*) and 9311 were susceptible (Table 2 and Figure 2).

### 2.4. An Improved Breeding Line Containing Pigm-1 Gene Was Obtained

In this study, S19-118 was selected and used as the maternal parent, and R20-4, a selected restorer line containing *Pigm-1*, was used as the male parent for hybridization. Then the hybrid F_1_ was backcrossed with S19-118, and a single strain containing the *Pigm-1* gene was selected and further backcrossed with S19-118 by combining molecular markers until BC_3_F_1_ was followed by selfing. Each generation was tracked by molecular markers and planting was continued for 3 generations until BC_3_F_9_. Other agronomic traits of the line were stable.

**Figure 3:**
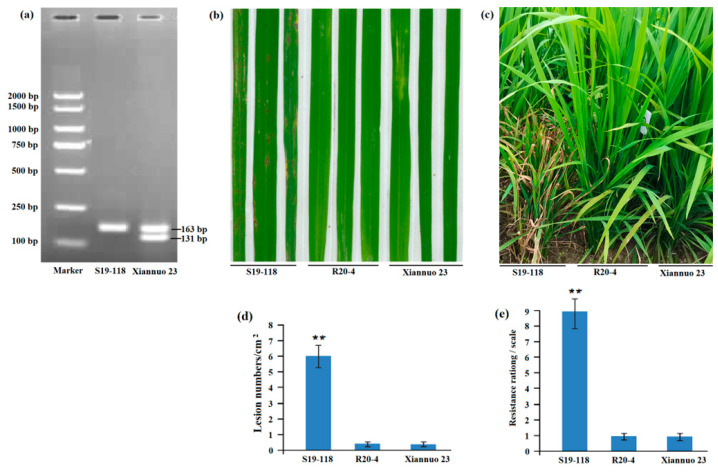
Detection of molecular markers in improved lines and phenotype of blast resistance. (**a**) The results of *Pigm-1* molecular marker detection showed that Xiannuo 23 contained *Pigm-1* that had two expected bands of 163 bp and 131 bp, while S19-118 without *Pigm-1* had only one band of 163 bp. (**b**) The greenhouse test results of the improved lines showed that S19-118 was susceptible to rice blast, while R20-4 and Xiannuo 23 were resistant to rice blast. (**c**) The field test results of the improved line showed that S19-118 was susceptible to, but R20-4 and Xiannuo 23 were resistant to, the rice blast variant Guy 11. (**d**) Lesion numbers per cm^2^ on the rice leaves (M ± SD, *n* > 10 leaves) in (**b**). (**e**) The incidence scores of the strains are based on the results in (**c**), the disease score of S19-118 is 9, while both R20-4 and Xiannuo 23 have a score of 1. Asterisks (**) indicate statistical significance (*p* < 0.01) determined by Student’s *t* test.

In the BC_3_F_9_, a line T22-23 with excellent comprehensive agronomic traits was selected and named Xiannuo 23. Finally, target molecular markers were used to genotype Xiannuo 23. The results showed that Xiannuo 23 contained the target gene *Pigm-1* (Figure 3a).

### 2.5. Analysis of Rice Blast Resistance of Xiannuo 23 Improved Line

In order to phenotypically identify the rice blast resistance of the created Xiannuo 23, we used the rice blast fungus Guy11 for resistance identification at the seedling stage. The results showed that the recipient parent S19-118 was susceptible to the disease, and the donor parent R20-4 and the improved line Xiannuo 23 were resistant to the disease (Figure 3b). To further evaluate the resistance of the improved line Xiannuo 23 under natural conditions, recipient parent S19-118, donor parent R20-4 and improved line Xiannuo 23 were all planted in the National Blast Resistance Identification Center in Shanghang County, Fujian Province. The test results were consistent with the laboratory test results, and the improved line Xiannuo 23 showed resistance to the disease (Figure 3c).

### 2.6. Analysis of Main Agronomic Characters of Xiannuo 23

**Figure 4:**
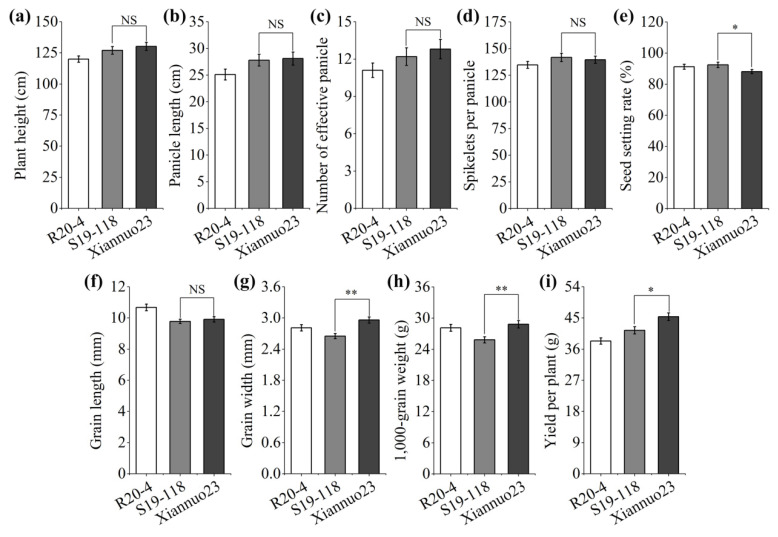
Comparison of main agronomic traits between parents and improved line Xiannuo 23. (**a**) Plant height. (**b**) Panicle length. (**c**) Number of effective panicle. (**d**) Spikelets per panicle. (**e**) Seed setting rate. (**f**) Grain length (**g**) Grain width. (**h**) 1,000-grain weight. (**i**) Yield per plant. NS: no statistically significant difference; * and ** show significant at *p* < 0.05 and 0.01 levels, respectively.

To further analyze the changes of other agronomic traits of Xiannuo 23 while improving resistance, we investigated and analyzed the agronomic traits of the donor parent R20-4, the recipient parent S19-118 and the improved line Xiannuo 23 at maturity. The results showed that, compared with the recipient parent S19-118, the improved line Xiannuo 23 had no change in plant height, panicle length, effective panicle, number of spiklets per panicle and grain length, but there were significant differences in seed setting rate, grain width and 1000-grain weight. Grain width and 1000-grain weight were significantly increased, while seed setting rate decreased. However, there was an increase in average yield per plant (Figure 4).

## 3. Discussion

### *3.1.* Pigm-1 Antispectral Analysis

Studies have shown that different alleles of rice blast may have different resistance spectra and resistance levels [8]. Several alleles have been identified at the *Piz* locus, such as *Pi2*, *Pi9*, *Piz-t* (*Pizh*), *Pigm* (*Pi50*) and *Pigm-1* [10,11,12,16]. Xie et al. [19] used 31 representative *M. oryzae* isolates from different regions to inoculate disease-resistant materials containing *Pi9*, *Pi2* and *Pigm* and found that Gumei4 (*Pigm*) was resistant to all the isolates tested, C101A51 (*Pi2*) was susceptible to eight isolates and 75-1-127 (*Pi9*) was susceptible to five isolates. Peng et al. [20] used 444 representative *M. oryzae* isolates from the Hunan Province to inoculate monogenic rice lines carrying the resistance genes *Pita*, *Pizt*, *Pikm*, *Pib* or *Pi9*. The result shows that both *Pi9* and *Pikm* conferred resistance to >75% of the tested isolates, while *Pizt*, *Pita* and *Pib* were effective against 55.63%, 15.31% and 3.15% of the isolates, respectively. Wu et al. [21] showed in a field inoculation that the resistance profiles of *Pi40* and *Pigm* were higher than *Pi2*, while *Pi2* was higher than *Pi9*. These results indicated that the resistance spectra of *Pigm* and *Pi40* were higher than those of *Pi9* and *Pi2*, and the resistance spectra of *Pi9* and *Pi2* were different in different regions or at different growth stages of rice.

The rice blast resistance genes *Pigm* and *Pigm-1* are both more broad-spectrum resistant in production [12,16], but it is not clear whether the resistance levels between them are different. In this study, 195 different blast fungi from the Fujian Province were isolated. The results showed that only one blast fungus JL-37 had differences in resistance to *Pigm* and *Pigm-1*, while the remaining 194 had no differences. In our previous studies, it was speculated that *Pigm* and *Pigm-1* had certain structural differences, and it was also speculated that this difference might be related to the evolution of rice blast fungus and the recognition of different pathogens by the R protein [16]. However, the identification of resistance proteins *Pigm* and *Pigm-1* with blast fungus JL-37 and the formation of a resistance mechanism still need to be further studied.

### *3.2.* Analysis of Pigm-1 Breeding and Utilization Prospects

Since cloning *Pigm-1*, we have created a series of new restorer materials using this gene. For example, using Minghui 63 as the receptor and Shuangkang 77009, which carries the resistance gene *Pigm-1*, as the donor, six new materials for resistance to rice blast were created [16]. Using restorer line R20 as the receptor and Shuangkang 77009, which carries the resistance gene *Pigm-1*, as the donor, four new materials for resistance to rice blast were generated [22]. Five new materials for resistance to rice blast and white leaf blight were prepared using Shuangkang 77009 with rice blast resistance gene *Pigm-1* and IRBBL23, with white leaf blight resistance gene *Xa23* as the donor [23]. Among these materials, the setting rate is above 90%. The allele of *Pigm-1*, *Pigm*, has been applied in rice disease resistance molecular breeding by more than 30 seed subsidiaries and breeding units in China, and new varieties have participated in regional trials and variety approval [12,16,24,25], which indicates that *Pigm-1* and *Pigm* have good application prospects in rice blast resistance breeding.

The latest study revealed that the PIBP4-Rab5a transport machine is involved in regulating the accumulation of the NLR protein PigmR in the cell membrane microregion, and the PigmR protein can activate the OsRac1 protein in the microregion, promote the production of reactive oxygen species and enhance the resistance of rice to blast disease [26]. The study identified a new NLR immune signaling pathway, providing a new perspective and target for PigmR disease resistance molecular breeding.

### 3.3. Further Study on the Relationship Between Pigm-1 and Waxy Genes

In this study, R20-4 containing *Pigm-1* was more than 90%. However, when Xiannuo 23 containing *Pigm-1* was cultivated with R20-4 containing *Pigm-1* as the donor, the seed setting rate was below 90% (Figure 4e). At the same time, in the process of creating glutinous rice materials, it was found that the seed setting rate of glutinous rice lines containing *Pigm-1* was generally lower than 90%, while the setting rate of glutinous rice lines without *Pigm-1* was relatively higher (>90%). Moreover, studies have shown that some rice blast *R* genes are linked to adverse agronomic traits often at the expense of yield [27,28]. Therefore, it is still uncertain whether the reason for the decreased seed setting rate in this study is the adverse linkage of *Pigm-1* and Waxy genes, and further research is needed to resolve this issue.

## 4. Materials and Methods

### 4.1. Isolation of Rice Blast Fungus

Rice blast fungus was isolated by referring to the method reported by Zhao et al. [29]. Fresh leaves with severe disease were collected from Shanghang County, Jianyang County and Jiangle County in the Fujian Province, and then brought to the experiment for single packet separation.

We collected blast fungi in areas with high blast incidence in the Fujian Province and isolated them by single packet isolation. Finally, we obtained 60 monospore blast fungi from Jiangle County (JL-1 to JL-60), 89 from Shanghang County (SH-1 to SH-89) and 46 from Jianyang County (JY-1 to JY-46, Table 2).

### 4.2. Rice Blast Fungus Incubation

For the internal resistance identification of rice blast, the inoculation method was improved with reference to the previous methods [30]. For spray inoculation, two-week-old rice seedling leaves were sprayed with a spore suspension (1 × 10^5^ spores/mL). The inoculated rice was placed in a moist chamber at 28 °C for 24 h in darkness and then transferred to another moist chamber with a photoperiod of 12 h under light. Leaves of 4-week-old rice seedlings were lightly wounded with a mouse ear punch, and 5 µL of spore suspension (5 × 10^5^ spores/mL) was added to the wound. The lesion size was measured after 5 days of incubation. Among the inoculated materials, there were 24 strains per material and 3 replicates were used.

The recipient parent S19-118, the donor parent R20-4 and the improved line Xiannuo 23 were incubated in the National Blast Resistance Identification Center in Shanghang County, Fujian Province on 18 May 2023. On 5 June 2023, the investigation and statistics of blast resistance of each strain at seedling stage were conducted. Plants were rated on a scale of 0–9 from the rice standard evaluation system (International Rice Research Institute 1996). Grades 0–3 were resistant, and grades 4–9 were susceptible.

### 4.3. Detection and Analysis of Pigm-1 and Pigm Functional Markers

**Figure 5:**
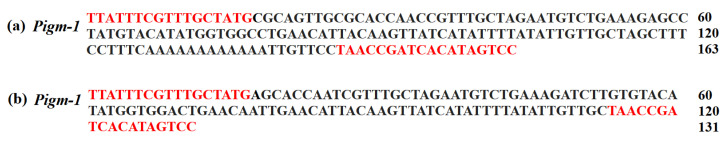
Primers designed for detecting the *Pigm-1* gene. The bases in red represent the location of the designed primer. (**a**) The *Pigm-1* specific primers amplified 163 bp bands. (**b**) The *Pigm-1* specific primers simultaneously amplified 131 bp bands.

The resistance function marker of *Pigm-1* refers to the marker developed by Yang et al. [16]. *Pigm-1*F: TTATTTCGTTTGCTATG; *Pigm-1*R: GGACTATGTGATCGGTTA. Specific primers were designed based on the *Pigm-1* sequence, and two bands of 163 bp and 131 bp were amplified with *Pigm-1* (Figure 5).

The resistance function marker of *Pigm* refers to the marker developed by Chen et al. [31]. T-*Pigm*-O-F: TAAGAATTGAGGTGGTTAGTTGAACGGAGA; T-*Pigm*-O-R: TTGCATGGCTCCACTACCCACTATAAG. For the extraction of genomic DNA from rice leaves, and the detection and analysis of molecular marker genes, refer to Yang et al. [16].

### 4.4. Investigation of Agronomic Traits

In July 2023, experimental materials, such as R20-4, S19-118, Xiannuo 23, 9311 (*Pigm-1*), 9311 (*Pigm*) and 9311, were planted in the experimental farm of the Rice Research Institute of Fujian Academy of Agricultural Sciences. The materials were all planted in the same plot, 3 replicates were set in each plot, 5 rows in each plot and 8 plants in each row. Field cultivation techniques are managed according to conventional cultivation techniques.

The main agronomic traits of rice, such as plant height, panicle length and seed setting rate, were investigated and analyzed with reference to Beena et al. [32].

## Figures and Tables

**Table 1:** Comparison of the main agronomical traits between 9311 (*Pigm-1*), 9311 (*Pigm*) and 9311.

Traits	9311 (*Pigm-1*)	9311 (*Pigm*)	9311
Plant height (cm)	115.4 ± 2.24 *	107.3 ± 2.14	106.8 ± 2.11
Panicle length (cm)	23.1 ± 0.34 *	20.7 ± 0.31	19.4 ± 0.30
Number of effective panicle	7.68 ± 1.06	7.91 ± 1.13	7.84 ± 1.19
Spikelets per panicle	197.6 ± 6.46 **	163.7 ± 5.96	160.8 ± 5.16
Seed setting rate (%)	91.06 ± 2.23	90.15 ± 1.92	88.85 ± 1.62
1000-grain weight (g)	32.13± 0.91	30.98± 0.81	31.16± 0.81
Grain length (mm)	9.75 ± 0.18	9.68 ± 0.21	9.71 ± 0.21
Grain width (mm)	3.26 ± 0.10	3.23 ± 0.10	3.24 ± 0.11

* and ** indicate the significance levels of the differences between 9311 (*Pigm-1*), 9311 (*Pigm*) and 9311 were revealed by the *t*-test at *p* < 0.05 and *p* < 0.01, respectively. The data were derived from the trial that was performed at the Fuzhou experimental station in October 2023.

**Table 2:** The results of indoor inoculation of single gene line 9311 (*Pigm-1*), 9311 (*Pigm*) and 9311 by *M. oryzae* isolates collected from different areas of the Fujian Province.

Name	9311 (*Pigm-1*)	9311 (*Pigm*)	9311	Name	9311 (*Pigm-1*)	9311 (*Pigm*)	9311
JL-1	R	R	S	SH-41	R	R	S
JL-2	R	R	S	SH-42	R	R	S
JL-3	R	R	S	SH-43	R	R	S
JL-4	R	R	S	SH-44	R	R	S
JL-5	R	R	S	SH-45	R	R	S
JL-6	R	R	S	SH-46	R	R	S
JL-7	R	R	S	SH-47	R	R	S
JL-8	R	R	S	SH-48	R	R	S
JL-9	R	R	S	SH-49	R	R	S
JL-10	R	R	S	SH-50	R	R	S
JL-11	R	R	R	SH-51	R	R	S
JL-12	R	R	R	SH-52	R	R	S
JL-13	R	R	S	SH-53	R	R	S
JL-14	R	R	S	SH-54	R	R	S
JL-15	R	R	S	SH-55	R	R	S
JL-16	R	R	S	SH-56	R	R	S
JL-17	R	R	S	SH-57	R	R	S
JL-18	R	R	S	SH-58	R	R	S
JL-19	R	R	S	SH-59	R	R	S
JL-20	R	R	S	SH-60	R	R	S
JL-21	R	R	S	SH-61	R	R	S
JL-22	R	R	S	SH-62	R	R	S
JL-23	R	R	S	SH-63	R	R	S
JL-24	R	R	S	SH-64	R	R	S
JL-25	R	R	S	SH-65	R	R	S
JL-26	R	R	S	SH-66	R	R	S
JL-27	R	R	S	SH-67	R	R	S
JL-28	R	R	S	SH-68	R	R	R
JL-29	R	R	S	SH-69	R	R	S
JL-30	R	R	S	SH-70	R	R	S
JL-31	R	R	S	SH-71	R	R	S
JL-32	R	R	S	SH-72	R	R	S
JL-33	R	R	S	SH-73	R	R	S
JL-34	R	R	S	SH-74	R	R	S
JL-35	R	R	S	SH-75	R	R	S
JL-36	R	R	S	SH-76	R	R	S
JL-37	S	R	S	SH-77	R	R	S
JL-38	R	R	S	SH-78	R	R	S
JL-39	R	R	S	SH-79	R	R	S
JL-40	R	R	S	SH-80	R	R	S
JL-41	R	R	S	SH-81	R	R	S
JL-42	R	R	S	SH-82	R	R	S
JL-43	R	R	S	SH-83	R	R	S
JL-44	R	R	S	SH-84	R	R	S
JL-45	R	R	S	SH-85	R	R	S
JL-46	R	R	S	SH-86	R	R	S
JL-47	R	R	S	SH-87	R	R	S
JL-48	R	R	S	SH-88	R	R	S
JL-49	R	R	S	SH-89	R	R	S
JL-50	R	R	S	JY-1	R	R	S
JL-51	R	R	S	JY-2	R	R	S
JL-52	R	R	S	JY-3	R	R	S
JL-53	R	R	S	JY-4	R	R	S
JL-54	R	R	S	JY-5	R	R	S
JL-55	R	R	S	JY-6	R	R	S
JL-56	R	R	S	JY-7	R	R	R
JL-57	R	R	S	JY-8	R	R	S
JL-58	R	R	S	JY-9	R	R	S
JL-59	R	R	S	JY-10	R	R	S
JL-60	R	R	S	JY-11	R	R	S
SH-1	R	R	S	JY-12	R	R	S
SH-2	R	R	S	JY-13	R	R	S
SH-3	R	R	S	JY-14	R	R	S
SH-4	R	R	S	JY-15	R	R	S
SH-5	R	R	S	JY-16	R	R	S
SH-6	R	R	S	JY-17	R	R	S
SH-7	R	R	S	JY-18	R	R	S
SH-8	R	R	S	JY-19	R	R	S
SH-9	R	R	S	JY-20	R	R	S
SH-10	R	R	S	JY-21	R	R	S
SH-11	R	R	S	JY-22	R	R	S
SH-12	R	R	S	JY-23	R	R	S
SH-13	R	R	S	JY-24	R	R	S
SH-14	R	R	S	JY-25	R	R	S
SH-15	R	R	S	JY-26	R	R	S
SH-16	R	R	S	JY-27	R	R	S
SH-17	R	R	R	JY-28	R	R	S
SH-18	R	R	S	JY-29	R	R	S
SH-19	R	R	S	JY-30	R	R	S
SH-20	R	R	S	JY-31	R	R	S
SH-21	R	R	S	JY-32	R	R	S
SH-22	R	R	S	JY-33	R	R	S
SH-23	R	R	S	JY-34	R	R	S
SH-24	R	R	S	JY-35	R	R	S
SH-25	R	R	S	JY-36	R	R	S
SH-26	R	R	R	JY-37	R	R	S
SH-27	R	R	S	JY-38	R	R	S
SH-28	R	R	S	JY-39	R	R	S
SH-29	R	R	S	JY-40	R	R	S
SH-30	R	R	S	JY-41	R	R	S
SH-31	R	R	S	JY-42	R	R	S
SH-32	R	R	S	JY-43	R	R	S
SH-33	R	R	S	JY-44	R	R	S
SH-34	R	R	S	JY-45	R	R	S
SH-35	R	R	S	JY-46	R	R	S
SH-36	R	R	S				
SH-37	R	R	S				
SH-38	R	R	S				
SH-39	R	R	S				
SH-40	R	R	S				

## Data Availability

Data are contained within the article.
