# Correction: Jin et al. Resistance Spectrum Analysis and Breeding Utilization of Rice Blast Resistance Gene *Pigm-1*. *Plants* 2025, *14*, 535

**DOI:** 10.3390/plants14213313

**Published:** 2025-10-30

**Authors:** Yidan Jin, Niqing He, Zhaoping Cheng, Shaojun Lin, Fenghuang Huang, Wenxiao Wang, Qingshun Q. Li, Dewei Yang

**Affiliations:** 1 Institute of Rice, Fujian Academy of Agricultural Sciences, Fuzhou 350018, China; jyd0121@163.com (Y.J.); heniqing430@163.com (N.H.);; 2 College of Agriculture, Fujian Agriculture and Forestry University, Fuzhou 350002, China; 3 Biomedical Sciences, College of Dental Medicine, Western University of Health Sciences, Pomona, CA 91766, USA

**Error in Figure**

In the original publication [1], there was a mistake in Figure 3c as published. Figure 3c was not the correct phenotypic picture of the test series. The corrected Figure 3c appears below. The authors state that the scientific conclusions are unaffected. This correction was approved by the Academic Editor. The original publication has also been updated.

## Figures and Tables

**Figure 3:**
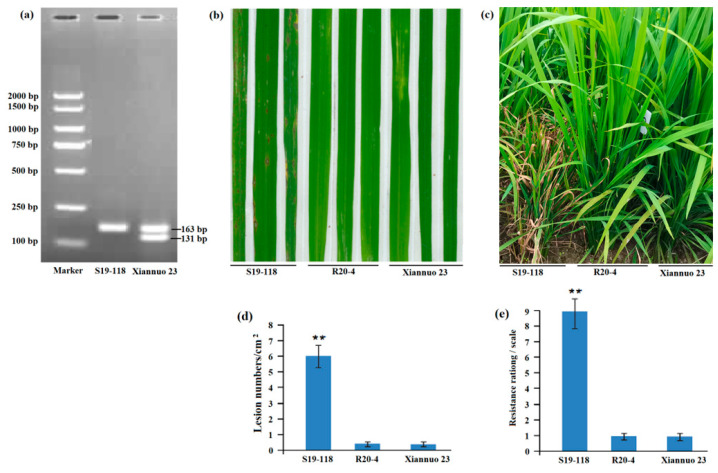
Detection of molecular markers in improved lines and phenotype of blast resistance. (**a**) The results of *Pigm-1* molecular marker detection showed that Xiannuo 23 contained *Pigm-1* that had two expected bands of 163 bp and 131 bp, while S19-118 without *Pigm-1* had only one band of 163 bp. (**b**) The greenhouse test results of the improved lines showed that S19-118 was susceptible to rice blast, while R20-4 and Xiannuo 23 were resistant to rice blast. (**c**) The field test results of the improved line showed that S19-118 was susceptible to, but R20-4 and Xiannuo 23 were resistant to, the rice blast variant Guy 11. (**d**) Lesion numbers per cm^2^ on the rice leaves (M ± SD, *n* > 10 leaves) in (**b**). (**e**) The incidence scores of the strains are based on the results in (**c**), the disease score of S19-118 is 9, while both R20-4 and Xiannuo 23 have a score of 1. Asterisks (**) indicate statistical significance (*p* < 0.01) determined by Student’s *t* test.
